# A drive for structure: A longitudinal qualitative study of the implementation of the Carer Support Needs Assessment Tool (CSNAT) intervention during hospital discharge at end of life

**DOI:** 10.1177/0269216320930935

**Published:** 2020-06-03

**Authors:** Alex Hall, Gail Ewing, Christine Rowland, Gunn Grande

**Affiliations:** 1 School of Health Sciences, University of Manchester, Manchester, UK; 2 National Institute for Health Research (NIHR) Collaboration for Leadership in Applied Health Research and Care Greater Manchester (CLAHRC GM), Salford Royal Foundation NHS Trust, Salford, UK; 3 Centre for Family Research, University of Cambridge, Cambridge, UK

**Keywords:** Caregivers, implementation science, needs assessment, palliative care, patient discharge, terminal care, qualitative research

## Abstract

**Background:**

Informal carers are essential in enabling discharge home from hospital at end of life and supporting palliative patients at home, but are often ill-prepared for the role. Carers’ support needs are rarely considered at discharge. If carers are less able to cope with home care, patient care may suffer and readmission may become more likely.

**Aim:**

To investigate the implementation of an evidence-based Carer Support Needs Assessment Tool (CSNAT) intervention to support carers during hospital discharge at end of life.

**Design:**

Longitudinal qualitative study with thematic analysis.

**Setting/participants:**

One National Health Service Trust in England: 12 hospital practitioners, one hospital administrator and four community practitioners. We provided training in CSNAT intervention use and implementation. Practitioners delivered the intervention for 6 months. Data collection was conducted in three phases: (1) pre-implementation interviews exploring understandings, anticipated benefits and challenges of the intervention; (2) observations of team meetings and review of intervention procedures and (3) follow-up interviews exploring experiences of working with the intervention.

**Results:**

Despite efforts from practitioners, implementation was challenging. Three main themes captured facilitators and barriers to implementation: (1) structure and focus within carer support; (2) the ‘right’ people to implement the intervention and (3) practical implementation challenges.

**Conclusions:**

Structure and focus may facilitate implementation, but the dominance of outcomes measurement and performance metrics in health systems may powerfully frame perceptions of the intervention and implementation decisions. There is uncertainty over who is best-placed or responsible for supporting carers around hospital discharge, and challenges in connecting with carers prior to discharge.

**What is already known about the topic?**

- Carers are essential in enabling discharge home from hospital at end of life and supporting palliative patients at home.
- Carers are often ill-prepared for this role, and support tends to focus on patient needs rather than carers’ own needs.
- The Carer Support Needs Assessment Tool (CSNAT) intervention is proven to be beneficial in home care, and has potential to be used in the context of hospital discharge.

**What this paper adds?**

- This paper identifies facilitators and challenges to implementing evidence-based carer support in the context of hospital discharge of palliative care patients.
- The dominance of outcomes measurement and performance metrics in health systems may frame practitioners’ perceptions of interventions and implementation strategies.
- There is uncertainty over which practitioners may be best-placed to facilitate carer support in the acute hospital setting, and practical challenges in providing carer support in hospital environments.

**Implications for practice, theory or policy**

- This paper reinforces the importance of conducting exploratory, qualitative work in the implementation of evidence-based interventions in new contexts.
- There are unresolved questions about the ‘fit’ of carers within health systems, and who is responsible for supporting them, which have implications for delivery of interventions like the CSNAT intervention in routine practice.

## Background

Most people nearing end of life would prefer to die at home.^1^ Informal carers (i.e. family/friends) are essential in enabling hospital discharge and supporting palliative patients at home, but are often ill-prepared for the role.^2^ Support for carers during hospital discharge at end of life is advocated in policy guidelines, but focuses on patient needs, and carers can often have unrealistic expectations of support available at home.^3^ If they feel less able to manage home care, patient readmission may become more likely.^4,5^ Carers supporting palliative patients at home face substantial demands upon their time, and upon their mental and physical health.^6–9^ In the coming decades, more people will be dying at home with increasingly complex care needs.^10,11^ Recent European work has highlighted the need for family carers to be recognised as part of the ‘unit of care’, but carer support often occurs ad hoc rather than being systematic within health systems.^12^ High-quality support for carers is therefore a growing imperative, but difficult to realise.

The Carer Support Needs Assessment Tool (CSNAT) intervention was developed with carers to provide an evidence-based, valid and feasible approach to assess and address carer support needs towards the end of life.^13,14^ Its person-centred approach is a change from usual practice, helping carers to lead the identification of their own support needs and supportive input, and explicitly acknowledging the difference in needs between patient and carer (Box 1).^15^ An Australian trial has shown that compared to the usual informal practices of identifying carers’ needs, the CSNAT intervention resulted in significant improvements in preparedness to care after discharge^16^ and reduced carer strain.^17^ The UK trial work has also shown improved outcomes in bereavement.^18^

**Box 1.** The Carer Support Needs Assessment Tool (CSNAT) intervention.

**Two components of the intervention: the ‘tool’ and the ‘approach’** The CSNAT intervention consists of a comprehensive tool comprising 14 broad support domains (seven relating to support enabling the carer to care for the patient, and other seven relating to preservation of the carer’s own well-being), integrated into a person-centred approach to assessment and support. This five-step ‘CSNAT Approach’ comprises (1) introduction of the tool to carers as a ‘conversation starter’; (2) time for the carer to consider the domains in which they need more support and prioritise those that are most important to them; (3) an assessment conversation with a practitioner in which the carer identifies their individual support needs in relation to the domains prioritised and what they would find helpful in meeting those needs. This enables (4) development of a shared action plan, which is recorded and (5) subsequently reviewed. **Broad domains and individual needs** The CSNAT domains are *broad areas* of support needs, for example, ‘managing your relative’s symptoms including giving medicines’ covers a wide range of carers’ support needs. The domains provide visibility about areas of support for carers and facilitate reflection. A person-centred approach is used to start a conversation with carers about *their specific, individual support needs*. The same domain will trigger different conversations about individual support needs with different carers. For example, one carer’s support need in relation to ‘managing your relative’s symptoms including medicines’ may be about understanding how to better manage a particular symptom, such as breathlessness or fatigue and information can be given to meet this need. For another carer, their need may be about worries about giving the patient certain medicines, like morphine, and so the supportive input required to meet this need will be quite different. The structure of the CSNAT (the tool itself), which has domains, not individual needs, as ‘items’ means that the CSNAT is not an outcome measure. Assessments over time can only indicate the broad domains where the carer has identified the need for more support, not their individual needs within those domains.
**The Care Act (2014) and the CSNAT intervention** In England, recent changes to legislation mean that local authorities have a duty to offer a carer assessment.^19^ This is a welcome development for carers generally. However, it does not address the complexity of caring for someone towards the end of life and specifically does not address support enabling carers to care for the patient. Furthermore, implementation appears to have been inconsistent, with around a quarter of carers providing palliative or end-of-life care having to wait more than 6 months for this assessment.^9^

CSNAT intervention implementation to date has occurred mostly in community and hospice settings. In this paper, we report on the implementation of the intervention at hospital discharge.

## Methods

### Aim

To implement a carer assessment and support intervention at hospital discharge and identify factors that helped or hindered implementation.

### Design

This study was a longitudinal qualitative exploration of implementation of the CSNAT intervention at hospital discharge. It forms part of a wider piece of work guided by the Medical Research Council’s complex interventions framework.^20^ Development work conducted in 2015 with carers and practitioners showed that the CSNAT intervention had the potential to facilitate conversations about the realities of end-of-life caregiving at home.^3^ Carers and practitioners recommended a two-stage approach to assessment and support, proposing an initial hospital assessment linked to post-discharge follow-up by community practitioners. They also felt that the intervention should be introduced early to carers, before the discharge process was fully set in motion. The present study was informed by these recommendations.

### Setting

The study was conducted from March–December 2018 within one National Health Service (NHS) Trust in England serving a large, socioeconomically and culturally diverse population.

### Population, sampling and recruitment

Participants were members of a hospital-based Supportive and Palliative Care Team (hereafter ‘hospital team’) and a Community Macmillan Team (hereafter ‘community team’). They were identified from the preliminary research^3^ and via senior nursing staff in the Trust, as being potentially well placed to initiate the intervention in the hospital and facilitate post-discharge follow-up. Some team members had taken part in the preliminary study, and therefore had some familiarity with the CSNAT intervention. All team members were eligible for participation. As the study focussed on practitioner experiences of working with the CSNAT intervention, no patients or carers were recruited.

### Training

Between March and May 2018, participants took part in two face-to-face training workshops. The first covered development of a service-level implementation strategy with a core group who would lead implementation (it is not necessary for all team members to attend this training). The second trained the full teams in how to use the intervention. Training was led by GE, with support from AH and CR. See http://csnat.org/training for further information on training content.

Practitioners identified a champion (a hospital team nurse) to lead implementation (e.g. development and communication of the implementation strategy and regular updates at team meetings). An administrator was given dedicated time to provide project support. Practitioners had freedom to fit the intervention into their daily practice (while maintaining its fidelity), and were encouraged to have ongoing reflection on the implementation process. The only stipulation made by the research team was that the process of carer assessment and support should start in the hospital.

### Data collection

Data collection occurred in three phases: (1) pre-implementation (May–July 2018) involved individual and group interviews to explore participants’ understandings of the intervention, anticipated benefits and practice challenges; (2) implementation (June–November 2018) involved observation of regular team meetings, and review of procedural documents associated with the intervention, to track implementation and to triangulate interview data^21^ and (3) follow-up (November–December 2018) involved second-round interviews to explore participants’ reflections on working with the intervention. Interview topic guides are provided as a supplementary file.

Interviews were conducted in participants’ workplaces, according to their availability, and were audiorecorded. Project implementation meetings were audiorecorded; field notes were written about team meetings and documents. All data were collected by AH, with support in group interviews from CR.

Practitioners also collected their own data: numbers of carers introduced to the CSNAT, assessment conversations and follow-ups by the community team. These descriptive statistics were passed to the research team at the end of the implementation period.

### Data analysis

Recordings were transcribed verbatim, handwritten notes typed up and imported into NVivo 12 software. Thematic analysis was conducted:^22^ AH led the process, conducting coding and developing themes. He regularly discussed ongoing interpretations with CR. Final codes and themes were checked by GE and GG, and interpretations discussed and agreed. Interpretations were also discussed with the hospital team champion during the production of a report for the NHS Trust.

### Ethics and consent

Ethics approval was granted by the University of Manchester Alliance Manchester Business School Panel in January 2018 (ref. 2018-3489-4690). All participants provided written consent.

## Results

All members of both teams were invited to participate, and none declined. In total, 17 participated, 13 from the hospital team (three consultants, nine nurses, one administration staff member) and four from the community team (all nurses). One consultant joined the hospital team shortly after study start, and two hospital team nurses left mid-study. All clinicians were palliative care specialists, educated to a minimum of degree level. Table 1 provides further details about participants.

**Table 1.** Participant information and involvement.

Team	ID	Role	Training: implementation workshop	Training: intervention workshop	First-round interview	Second-round interview
Community	CN01	Nurse	•	•	Unavailable	Unavailable
	CN02	Nurse	•	•	Community group	Community group
	CN03	Nurse	•	•	Community group	Community group
	CN04	Nurse	•		Community group	Unavailable
Hospital	HN05	Nurse		•	Group A	Group C
	HN06	Nurse		•	Group A	Had left team
	HN07*	Nurse	•	•	Group A	Group B
	HN09	Nurse		•	Group B	Had left team
	HN11	Nurse		•	Group B	Group B
	HN13	Nurse		•	Group C	Group D
	HN14	Nurse		•	Group C	Group A
	HN17	Nurse			Individual	Group A
	HM15	Matron		•	Individual	Group D
	HA10	Project Officer	•	•	Group B	Group A
	CT08	Consultant		•	Group A	Unavailable
	CT12	Consultant		•	Group B	Group A
	CT16	Consultant			Had not joined team	Group C

* Project champion.

We conducted nine group interviews and two individual interviews (see Table 1), lasting 42–84 min.

Overall, implementation proved challenging, despite the efforts of the practitioners. The hospital team introduced the CSNAT intervention to 12 carers, six of these carers self-completed the tool and three had assessment conversations. These three were handed over to the community team for follow-up, with one being reassessed.

To understand the context, we summarised implementation processes employed by practitioners to support practice change. In our thematic analysis, we developed three overarching themes: (1) structure and focus within carer support; (2) the ‘right’ people to implement the intervention and (3) practical challenges for implementation. Each will be explored to highlight implementation successes and challenges. Italics indicate verbatim quotations. Identification numbers are explained in Table 1. Data source is also represented (e.g. i1 = first-round interview; i2 = second-round interview).

### Context: implementation processes for practice change

This project involved a change in practice: implementation of a new intervention. It occurred within the organisational context of an NHS Trust in which practice change normally is supported by a systematic and largely quantitative quality improvement methodology, said to be ‘quite a structured approach, which is quite nice’ (HN06; i1). To set boundaries around a new implementation project, practitioners decided to set criteria for offering the CSNAT intervention, feeling that ‘anything else would have been out of control and unmanageable, and we would’ve fallen at the first hurdle’ (HN07; i2). However, they later reflected they may have set their criteria too narrow, contributing to the low number of assessments. Some practitioners struggled with the concept of an implementation study exploring their experiences, rather than an intervention study evaluating carer outcomes. This contributed to concerns about getting implementation ‘wrong’, summed up by the champion’s reflection that ‘the biggest challenge for me leading on the implementation was getting people to understand that this was as much a learning exercise for us. . . [colleagues] asked me for a lot of answers to fix problems that hadn’t actually occurred or might not even occur’ (HN07; i2).

Practitioners worked hard to keep the intervention visible in daily routines, incorporating project updates in team briefs, holding regular project-specific meetings and using visual reminders. They also developed feedback forms for themselves to capture experiences of intervention use. Observations showed that in time-pressured team meetings, updates were about upcoming assessments, rather than completed assessment conversations. Some practitioners felt that quantitative feedback would have been useful, reflecting that ‘we’ve never really thought about feedback in a structured, data analysis mind-set’ (HM15; i2) and that it may have been useful to know ‘how many CSNATs have we achieved this week, this month?’ (HN13; i2). At project outset, some highlighted the importance of discursive reflection, especially considering the peripatetic nature of specialist palliative care which meant that ‘. . . most of us are lone workers most of the time’ (HN14; i1). However, observations indicated a lack of qualitative discussion about experiences of implementation, and some practitioners later reflected that ‘we need to talk about it more’ (HN05; i2).

### Theme 1: structure and focus within carer support

The structure and focus of the CSNAT tool helped facilitate implementation as practitioners felt it offered a more comprehensive assessment that gave them more confidence in assessing carers’ support needs. It also helped maintain a focus on carers’ needs over time, as one practitioner reflected ‘quite a few things we got resolved right there and then on that day. But it also kept in mind those other issues that we hadn’t yet resolved to make sure that we brought those back up again’ (HN11; i2). More broadly, the staged delivery of the intervention appeared to lead to a more visible record of carer support that could be used to effect change. This was exemplified in one situation regarding a carer complaint, where

[the carer] said he’d told people but nothing had been done. So because it’s in writing as well it gets a little bit more formal, people take more notice of it. Put a copy in the notes, the ward team could see what the issues were. But also it [gave] me some clout to say well, these are his feelings, this is what he’s struggling with on the ward, and we need to do something about this. (HN07; i2)

There was debate about whether foregrounding carers’ needs presented a threat or an opportunity. Some practitioners worried that ‘it might raise [carers’] expectations too much, that there’s going to be some all singing, all dancing service, that’s going to rush in and look after them’ (CN01; i1). Others suggested that ‘it will finally give us some evidence to say, ‘these are the challenges we’ve got to support carers’’ (HN07; i1) or ‘evidence to show that this is what we do’ (CN03; i2) when supporting carers. This evidencing seemed to be very desirable; practitioners highlighted that carers tend to be viewed within healthcare systems as ‘part of the patient, as opposed to their own individual person’ (HN13; i1), and with no dedicated recording system for carers, time spent with carers is bundled into patient statistics. One nurse explained that ‘if you spent 20 minutes with the patient and their symptoms were very well managed, but then the relative spoke to you for 40 minutes, it would all be down as just a 60 minute contact. And you’d tick family support, symptom management, admin, for the whole thing’ (HN06; i1).

The structure of the CSNAT tool was felt to have the potential to address these challenges, but this seemed to be predicated on perceiving it as an outcome measure rather than as one component of a conversational intervention. Some felt that ‘because it’s structured, we can measure our intervention. . . currently [it] is a bit difficult to quantify’ (CT08; i1). Similarly, others suggested that a follow-up assessment in the community could enable repeat measurement and seeing ‘if they were mentioning [CSNAT domain] one, two, three in hospital and then now mentioning seven, eight, nine, what’s changed?’ (CT12; i1). Several practitioners wondered whether carers would want to use ‘paperwork’ (HN14; i1) to consider their needs, whether carers would ‘fill it in truthfully’ (CN02; i1), or expressed concerns about ‘bias[ing] their answers’ (HN06; i1) if they supported carers in completing the tool itself. Perceptions of the tool were thus at least partly framed by the prevalence of standardised outcome measures in healthcare, particularly the use of the Integrated Palliative Care Outcome Scale (IPOS; a patient-reported outcome measure). This appeared to have partly influenced unsuccessful implementation strategies, such as leaving copies with carers in the hospital that were never returned; as one practitioner reflected, this approach ‘never really worked [with the IPOS], so I don’t know why I did it [with the CSNAT]’ (HN13; i2).

Despite the appeal of gathering specific evidence about carer support needs or practitioner input, there was an enduring generic implementation challenge of where to record this in the absence of a separate recording system for carers.

### Theme 2: the ‘right’ people to implement the intervention?

Practitioners had mixed views on whether they were the ‘right’ people to be implementing given their positions within the health system. The community team felt that they had a better understanding of carers than other community health professionals, for example, ‘GPs. . . don’t always recognise the needs of the family members, because they’ve known them, and it sometimes takes a fresh set of eyes. . . for you to go in and think, ‘well they’re clearly struggling*’*’ (CN04; i1). Some of the hospital team reflected that they were ‘probably in one of the better positions to do it, because we allocate our time for things like that. . . and we follow patients through. . . we’re covering all wards’ (HN07; i2). However, others thought that ‘our [patient] turnover’s quite rapid, so I suppose that poses the question, is the acute setting the most appropriate setting to be implementing [the CSNAT intervention]?’ (HN14; i2). Some suggested that other practitioners would be well placed to support carers in the community, including district nurses because ‘they’re in those patients’ homes all the time’ (HN06; i1).

There were also contradictions about perceived skills and experience required for successful implementation. Many emphasised that their role as specialist palliative care practitioners was a facilitator, citing their advanced communication skills training and experience with difficult conversations. Some felt that ‘some of the things we’re asking carers to consider [via the CSNAT intervention] is actually no different than what would’ve been done anyway’ (HN11; i2), and wondered ‘if we roll that out to another service, who perhaps hasn’t got the same communication skills that we’ve got. . . I would envisage that would be an awful lot more problematic’ (HN07; i1). However, others suggested that lower-grade care assistants, who ‘would need foundational level communication skills’ (CN03; i2) might be better placed to take on the intervention. There were also suggestions senior NHS leadership might look to the voluntary sector to provide carer support, arguing ‘we’re paying you [the clinicians] money to go and look after patients, we’ll get voluntary sector to do this bit’ (CN03; i2).

### Theme 3: practical challenges for implementation

Participants anticipated and subsequently reflected that the workload in implementing the intervention was manageable. The role of the hospital team administrator was deemed a crucial facilitator; the administrator suggested that ‘at the moment while it is a small cohort it’ll be fine’ (HA10; i1), but wondered about future sustainability if implementation were expanded.

There were a number of practical challenges to implementation in the hospital. These included finding time and space to introduce the intervention to carers. Some examples seemed linked to the uncertainty of illness trajectories in palliative care; there was a sense of too early, where the patient was acutely unwell and practitioners deemed that the carer was not ready to focus on themselves, or too late as the patient was about to be discharged or had died. Observations from team meetings highlighted the extent of this challenge, for example, when one nurse (HN11) reported that ‘she had tried four times to catch the family to introduce [the CSNAT]. . . The fifth time she tried, she found the patient had died’ (field notes from multidisciplinary team meeting, 24/7/2018). Acute carer distress seemed sometimes to hinder implementation because ‘it’s difficult to even give psychological support, let alone then try and take it into an assessment of their needs. . . I kind of went with the intentions of bringing [CSNAT] up, and then it just never occurred’ (HN13; i2).

There were generic challenges in ensuring privacy; one nurse referred ironically to ‘pull[ing] those soundproof curtains around’ (HN17; i2) to conduct a conversation with a carer at the patient’s bedside. There were also perceived tensions between carer support and patient confidentiality. One example highlighted a carer who was not offered an assessment because the patient had previously stated she did not want practitioners to discuss any aspect of her care. The practitioner reported that she found this difficult to reconcile with the carer focus of the CSNAT intervention, as ‘it’s almost like [the patient]’s consenting for somebody else. . . when actually it’s not her needs we’re looking at, we’re looking at the needs of her family’ (HN17; i2).

Despite low numbers of assessments carried out, participants reflected positively that their experiences had ‘challenged some of our previous ideas about what sort of support we’re offering’ (HN13; i2) and had ‘made me think about assessing [carers] more, than focusing on the patients’ (HN05; i2).

## Discussion

We worked with specialist palliative care practitioners in hospital and community settings of one NHS Trust in England, to implement a carer assessment and support intervention to support carers of patients during hospital discharge at end of life, and identify factors that helped or hindered implementation. Overall, despite practitioner efforts, implementation proved challenging. Our study revealed three main findings: (1) an emphasis on structured approaches to work, dominance of outcomes measurement and performance metrics which framed practitioners’ perceptions of the intervention and their implementation decisions; (2) contradictions in the extent to which specialist practitioners felt that they were the ‘right’ people to implement the intervention and (3) practical implementation challenges of the hospital context.

We have previously examined factors affecting the implementation of carer assessment and support more broadly within end-of-life care.^23–25^ To better understand particular challenges encountered in this hospital context, we draw upon relevant aspects of the Nonadoption, Abandonment, Scale-up, Spread and Sustainability (NASSS) framework.^26^ This framework suggests that sustaining implementation may be very difficult in contexts in which health conditions are unpredictable, where the intervention does not directly measure changes in health condition, and where the intervention does not readily align with prevailing organisation and system beliefs, including what counts as ‘high-quality’ evidence. These were all challenges within the current project.

Unpredictable illness trajectories combined with substantial psychosocial issues in palliative and end-of-life care presented multiple implementation challenges in connecting with carers prior to hospital discharge. Practitioners perceived that the intervention could bring structure to carer support, provide evidence of carers’ support needs and quantify hidden work involved in supporting carers. These views facilitated implementation, but were also problematic because they revealed a belief that the CSNAT might be used as an outcome measure, when it is not designed for this (Box 1). Implementation thus appeared challenging within a culture which emphasises outcome measurement and performance metrics, and also where recording systems are solely patient-focused and lack ability to facilitate this kind of analysis of carer support.

The NASSS framework proposes that implementation may be more difficult long-term if there is lack of ongoing sense-making and reflection. The CSNAT intervention training foregrounds need for regular discursive reflection. However, while there were some reflective project meetings between core hospital team members and the community team, the greater focus overall was on numbers of CSNAT assessments, with a lack of overt reflective discussion among teams about implementation experiences. This may relate to lack of time in a busy clinical environment, but it is also possible that more discursive approaches to reflection were less compatible with the more quantitative practice-change methodology that practitioners were familiar with.

The NASSS framework suggests that implementation may be more difficult when the intervention requires substantial work to build a shared vision, does not align well with existing skills, routines and pathways of care and involves significant changes in practice. There was a shared project vision and valuing of carer assessment and support, which facilitated engagement in implementation work. However, the contradictions in whether practitioners felt they were the ‘right’ people to implement suggested some challenges in alignment with existing skills, routines and pathways. The hospital team administrator’s role in collating intervention information was deemed crucial in supporting the project champion, but raised questions about the potential for wider roll-out in the absence of this dedicated support. Other challenges generic to the hospital environment (lack of privacy; concern about patient confidentiality) are also important, because the intervention is designed to facilitate more comprehensive discussions with carers that go beyond the usual ‘and how are you?’ corridor conversations. More fundamentally, it presents a change in practice, moving away from practitioners’ perceptions of carers ‘not coping’, towards supporting carers to identify and express their own support needs and discuss potential solutions. Some practitioners initially felt discussions enabled by the intervention would be similar to what they already did, and perhaps did not fully grasp the changes to practice; for example, they still took the lead in judging when carers would be ready for the intervention. Although practice change is covered in the intervention training, it may be that revision is necessary to clarify principles and messages. However, further support with practice change requires input at the organisational level, posing added challenges for implementation by two small teams within a very large organisation. Nevertheless, practitioners largely reflected positively that their experiences had challenged some of their assumptions about support they provide to carers, and intended to continue to implement the intervention.

### Strengths and weaknesses

This was a small exploratory study within one NHS Trust in England. However, the dominance of quantitative approaches to practice is likely to prevail across health services nationally, and elsewhere, thus posing challenges for implementation and scale-up of more qualitative interventions. The nature of the project as an external piece of research may have partly framed practitioners’ implementation decisions and influenced what they considered possible. As adaptation is key to embedding interventions,^26^ use of an implementation protocol may have been beneficial: one that retained the five-staged approach for intervention fidelity but also promoted customising to the implementation setting, thus providing flexibility to promote embedding in practice. Nevertheless, there are enduring questions about the ‘fit’ of carer support interventions within health systems, illustrated by a lack of clarity of responsibility for carers, an absence of routine recording of carer details and a lack of consistent assessment of their support needs.^25^ These challenges need to be resolved if person-centred carer assessment and support is to be implemented more widely in hospital care.

## Conclusion

Carers are essential in enabling discharge home from hospital at end of life and supporting palliative patients at home, but are often ill-prepared for the role. The CSNAT intervention is proven to be beneficial in home care, and has potential to be used in the context of hospital discharge. This paper highlights facilitators and challenges to implementing the intervention in the acute hospital context, and reinforces the importance of conducting exploratory, qualitative work in implementation of evidence-based interventions in new contexts.

## Supplemental Material

CSNAT_Hosp_Discharge_Study_Int_Fg_schedules_v1-0 – Supplemental material for A drive for structure: A longitudinal qualitative study of the implementation of the Carer Support Needs Assessment Tool (CSNAT) intervention during hospital discharge at end of life Click here for additional data file. Supplemental material, CSNAT_Hosp_Discharge_Study_Int_Fg_schedules_v1-0 for A drive for structure: A longitudinal qualitative study of the implementation of the Carer Support Needs Assessment Tool (CSNAT) intervention during hospital discharge at end of life by Alex Hall, Gail Ewing, Christine Rowland and Gunn Grande in Palliative Medicine
