# Supplementary material for: A drive for structure: A longitudinal qualitative study of the implementation of the Carer Support Needs Assessment Tool (CSNAT) intervention during hospital discharge at end of life
Source: Palliat Med. 2020 Jun 3;34(8):1088–96. doi: 10.1177/0269216320930935 (PMC7388143; doi:10.1177/0269216320930935)
Supplement: CSNAT_Hosp_Discharge_Study_Int_Fg_schedules_v1-0 – Supplemental material for A drive for structure: A longitudinal qualitative study of the implementation of the Carer Support Needs Assessment Tool (CSNAT) intervention during hospital discharge at end of life [file CSNAT_Hosp_Discharge_Study_Int_Fg_schedules_v1-0.pdf]

The CSNAT Hospital Discharge Study
Interview/focus group schedules

Pre-CSNAT Implementation

Introductory explanation: We know that carers play a vital role in supporting patients in end-of-life care. We know that carers have their own support needs, and we want to gain an understanding of how identifying their needs and supporting them currently 'fits' within hospital systems. We want to explore this in terms of practitioners' individual practice, and at the service level through the work of the palliative care/community nursing teams.

Practitioner-level current practice

- Thinking first of all about your own practice, when you are referred patients:
    - What do you feel your role is in relations to carers?
    - Are carers ever referred to you, out with referral of patients?
    - How do you currently become aware of carer support needs?
    - What is your role in relation to delivery of support to patients and carers while in hospital?
    - What happens when the patient and carer are being discharged? What do you feel your role is in preparing the carer for caregiving at home?
- Thinking about the CSNAT intervention and its use in practice:
    - How do you think the CSNAT intervention differs from your current practice?
        - Will it enhance your practice – in what way?
        - Will it detract from your practice – in what way?
    - What challenges do you expect to encounter whilst using the CSNAT intervention?
        - How do you plan to address these challenges?

Service systems and processes currently in place

- Thinking about service-level systems and processes currently in place:
    - To what extent is your service constructed around carers as well as patients?
    - How is identification of carer needs currently accommodated within staff workloads and systems?
    - What do you feel your role is as a service in preparing the carers for discharge and for caregiving at home?
    - Do you feel your service delivers support to carers and in what way?
    - What are the visiting patterns to patients and carers?

- Thinking about facilitation processes employed for changes in practice:
    - How has your service implemented changes in practice in the past?
    - Have the methods used been successful? If not, why do you think this was?
    - How have these experiences influenced your planned approach to implementing the CSNAT intervention?

<u>Post-CSNAT Implementation</u>

Practitioner-level understanding of the CSNAT intervention and its use in practice

- How are you currently using the CSNAT intervention in your work?
- Have you found that using the CSNAT intervention in your practice has worked well – in what ways?
- Have you had problems in using the CSNAT intervention in your practice – in what ways?
    - Were these problems anticipated or unexpected – in what ways?
    - How did you attempt to address these problems, and were you successful?
- How does using the CSNAT intervention differ from your previous practice
    - Does it enhance your practice - in what way?
    - Does it detract from your practice – in what way?
    - What helps or hinders its use in your everyday practice?
    - Do you feel motivated about continuing to use this intervention - in what way?
        - If no, explore further about views of using / not motivated to use

Service-level adjustments for implementing the CSNAT intervention

- Thinking about service (or broader organisational) processes that have facilitated implementation of the CSNAT intervention:
    - What changes or modifications have been made to recording systems in relation to carer assessment?
    - Have there been changes to work patterns?
        - If so, in what way?
        - If not, how is use of the CSNAT intervention accommodated within workloads?
    - What are the external demands on your team that have competed with work on implementing the CSNAT intervention?
    - What activities has your service engaged in to promote implementation of the CSNAT intervention, to engage staff with this new approach to practice?
    - What methods of sustainable change management were used within your service to facilitate the shift to a person-led approach to carer assessment and support?
    - Which do you feel have been most useful – and why?
